# Epigenetic modification and BRAF gene mutation in thyroid carcinoma

**DOI:** 10.1186/s12935-021-02405-w

**Published:** 2021-12-19

**Authors:** Guo Huang, Juan Chen, Jun Zhou, Shuai Xiao, Weihong Zeng, Jiliang Xia, Xi Zeng

**Affiliations:** 1 Hengyang Medical College, University of South China, Hengyang, 421001 Hunan China; 2 Key Laboratory of Tumor Cellular and Molecular Pathology, College of Hunan Province, Cancer Research Institute, University of South China, Hengyang, 421001 Hunan China; 3 Department of Radiotherapy, The Second Affiliated Hospital, Hengyang Medical College, University of South China, Hengyang, 421001 Hunan China

**Keywords:** Thyroid Carcinoma, Epigenetics, BRAF, Treatment

## Abstract

Thyroid cancer remains the most prevailing endocrine malignancy, and a progressively increasing incidence rate has been observed in recent years, with 95% of thyroid cancer represented by differentiated thyroid carcinomas. The genetics and epigenetics of thyroid cancer are gradually increasing, and gene mutations and methylation changes play an important roles in its occurrence and development. Although the role of RAS and BRAF mutations in thyroid cancer have been partially clarified,but the pathogenesis and molecular mechanisms of thyroid cancer remain to be elucidated. Epigenetic modification refer to genetic modification that does not change the DNA sequence of a gene but causes heritable phenotypic changes in its expression. Epigenetic modification mainly includes four aspects: DNA methylation, chromatin remodelling, noncoding RNA regulation, and histone modification. This article reviews the importance of thyroid cancer epigenetic modification and BRAF gene mutation in the treatment of thyroid cancer.

## Introduction

Thyroid carcinoma (TC) is one of the most common endocrine malignancies. The incidence rate of malignant tumors in women is 4%, although with the yearly increase in the incidence rate, TC is expected to become the third largest form of cancer in women by 2030 [1, 2]. Over the past 10 years, with the advent of diagnostic models combined with ultrasound and fine needle punctures, the diagnosis of particularly micro-TC in particular increased. Currently available treatment packages for TC include surgical treatment, ^131^I treatment, local radiotherapy, radiofrequency ablation, and targeted treatment. TC patients exhibit a high survival rate (~ 98%)between 5 and 10 years, and the diagnosis of the cancer subtype is highly correlated with patient’s age [3]. Thyroid papillary cancer (PTC) and follicular thyroid cancer (FTC) are both caused by follicular epithelial cells and classified as differentiated thyroid cancer (DTC) and the less common Hurthle cell carcinoma [4]. Myelin-like thyroid cancer (MTC) originates from parathyroid C cells, with an incidence rate of approximately 5%–10%. Patients with distributive MTC often have larger tumors and are prone to advanced lymph node metastasis; moreover, the overall prognosis of MTC is worse than that of DTC [5]. Anaplastic thyroid cancer (ATC), produced in thyroid follicle cells, is one of the most invasive solid tumors in humans. The clinical prognosis of ATC is characterized by invasive local diseases, high metastasis rates, and rapid fatal clinical outcomes, with almost all patients dying within six months [6].

Epigenetics refers to regulating the gene expression levels through DNA methylation, histone modification, and RNA methylation without changing the sequence of DNA nucleotides, which changes the genetic information [7]. DNA methylation is a form of chemical modification that can change the genetic information without changing the DNA sequences. It is also the most widely studied epigenetic modification among plants and animals and plays a key role in their development, differentiation, and reproduction. DNA methylation occurs when DNA-methyltransferase (DNMT) is added to cytosine residues in CAG dinucleotides. These CAG dinucleotides are occasionally enriched on an island called CpG [8]. DNA methyltransferases (DNMT3A, DNMT3B, and DNMT3L) are mainly responsible for establishing genome site-specific DNA methylation models and play an important role in gene regulation and animal development. DNMT3A mutations increased significantly and showed poor prognosis in low-differentiated thyroid cancer (LDTC) patients. DNMT3A maintains the methylation state of the genome and can be used as a latent biomarker or therapeutic target for the prognosis and treatment of thyroid cancer [9]. In perpetochemical thyroid follicle cells, knocking out DNMT3B promotes SLC34A2 expression and a significant negative correlation is observed between DNMT3B and SLC34A2, which means that the expression of SLC34A2 is mediated by promoter methylation induced by the methyl transfer enzyme DNMT3B [10]. Lowering LINC00313 inhibits ALX4 methylation, inhibits the AKT/mTOR signalling axis, and inhibits proliferation, migration, invasiveness, and epithelial mesenchymal transitions (EMT) [11]. Histone acetylation is regulated by histone acetyltransferase (HATS) and histone deacetylation enzymes (HDACs) and participates in the regulation of gene expression. The biological functions of HDACs include physiological processes such as transcription regulation, metabolism, angiogenesis, DNA damage response, cell cycle, apoptosis, protein degradation, and immunity [12]. Compared to normal tissue, the levels of H3 histone acetylation at K18 residues in FA, PTC and FTC are higher, while there are no modifications in undifferentiated thyroid carcinomas (UC) are not observed. As a result, the level of H3K18 acetylation in UC is lower than that in DTC. These data show that H3 acetylation levels on the K18 residue decrease during the progression of thyroid tumours [13]. RNA methylation modification accounts for more than 60% of all RNA modifications, with m6A being the most common modification on advanced miRNA and lncRNA. Mir-181a promotes the growth of the cancer gene S100A2 and thyroid papillomastic cancer through the expression of the mediated histone demethylating enzyme KDM5C [14]. The 12 CpG sites in the PTC tissue located on the miR-204 promoter were reduced by hypermethylation, which promoted an increase in the expression of the target gene TRPM3, which may be associated with tumour sinvasion, lymph node metastasis, and BRAF^V600E^ mutations [15].Long noncoding RNA TNRC6C-AS1 promotes the methylation of STK4 through the Hippo signalling pathway, thereby suppressing apoptosis and autophagy in thyroid cancer cells [16]. m6A function is mainly determined by methylation transferases (METTL3, METTL14, TAP, RBM15, RBM15B, HAKAI, VIRMA, and ZC3H13), dimethylation enzymes (FTO and ALKBH5), and reading proteins (YTHDF1-3, YTHDC1-3, HNRNPA2B1, and eIF3) [17]. METTL3 may accelerate the progression of thyroid cancer by inducing TCF1 mRNA methylation by activating the Wnt pathway [18]. Therefore, this paper reviews the development of epigenetics and TC.

**Fig. 1:**
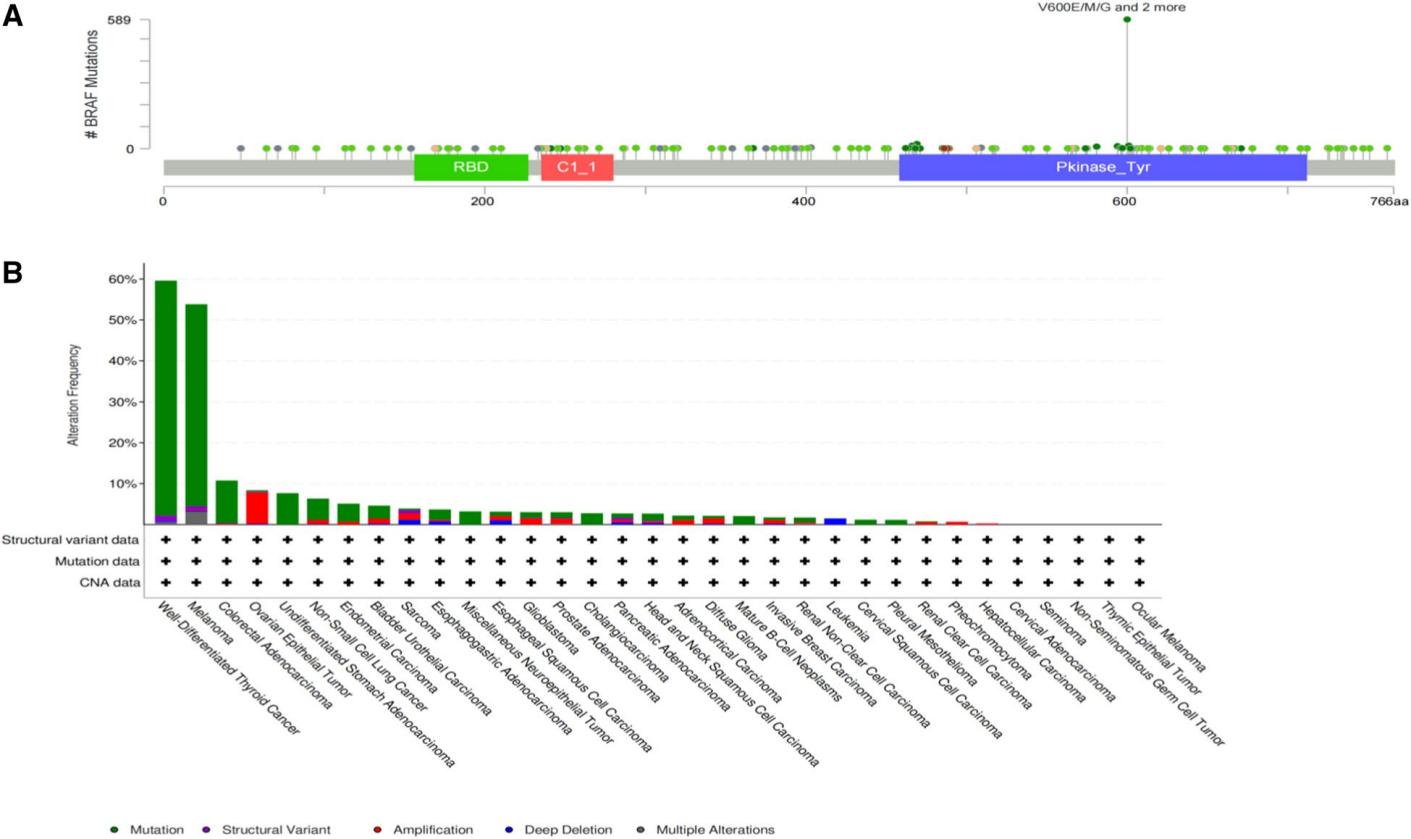
BRAF gene mutation sites and mutations in pan-cancer. **A** BRAF gene mutation site. **B** BRAF gene mutationin in pan-cancer, and the BRAF gene exhibits significant mutations in thyroid cancer

The BRAF gene, located on human chromosome 7 and encodes the serine/threonine protein kinase of the RAF family. It is an important part of the RAS-AF-MEK-ERK/MAPK signal transduction pathway and plays an important role in cell division, proliferation, and transformation [19]. We first obtained the gene mutation site and mutation status of BRAF in different tumours through the cBioPortal (https://www.cbioportal.org/) website. BRAF^V600E^ is located in the 15th exon of the BRAF gene. Its mutation is the T1799A point mutation in this exon, which changes its coded product, resulting in the substitution of valine (V) by glutamate (E). In the BRAF^V600E^ mutation, the cell cannot complete normal apoptosis, which further triggers the occurrence of tumours (Fig. 1A) [20]. BRAF^V600E^ mutations can be detected in TC, melanoma, colon cancer, ovarian serous cancer, malignant melanoma, and non-small-cell lung cancer (Fig. 1B). However, the use of BRAF gene mutations alone for TC diagnosis has certain limitations, and the occurrence of tumors also involves multiple genes. Therefore, we predicted the prognosis of patients with TC by combining TC diagnoses with other related mutant genes.

## Tumour suppressor gene methylation and BRAF gene mutation in TC

PTC is the most common thyroid malignancy, with BRAF and RAS gene mutations and RET/PTC rearrangement representing the main causes. The RAS-associated domain family protein 1 (RASSF1A) gene has been confirmed in more than 30 types of cancers and is the most common anticancer gene because of the silencing caused by the high methylation of this gene promoter, resulting in increased tumours invasion capacity. According to the literature, in early thyroid follicular carcinoma, the methylation of the RASSF1A gene promoter is inversely related to the BRAF gene mutation during the occurrence of thyroid tumours [21]. RASSF1A exhibits increased methylation in multiple lesions, epidural invasions, and lymph node metastasis, leading to tumour progression [22, 23]. RASSF1A promoter methylation is closely related to advanced thyroid cancer and elderly patients [24]. The initiation of the P16 and RASSF1A genes may lead to a risk of thyroid papilloma cancer [25]. RASSF1A as a clinical diagnositic factor for thyroid cancer still needs further verification.

Solute carrier family 5 (sodium/monocarboxylate cotransporter), member 8(SLC5A8) is a sodium-transporting protein that inhibits various solid tumors through methylation and has been observed in colon, stomach, lung, brain, and thyroid cancers [26]. Point mutations of the BRAF gene, such as serine/suline kinase, are most common in PTC. Approximately 70% of TCs have BRAF gene point mutations, and BRAF^V600E^ mutations in PTC patients are associated with invasive tumour phenotypes and increased risk of tumour recurrence, as the early mutations of the driving gene BRAF can alter the epigenetics of thyroid tumour tissue [27]. In addition to BRAF^v600E^ mutations in plasma, the methylation statuses of SLC5A8 and RASSF1A also act as good indicators for the identification of PTC and thyroid nodule cases [28].

**Fig. 2:**
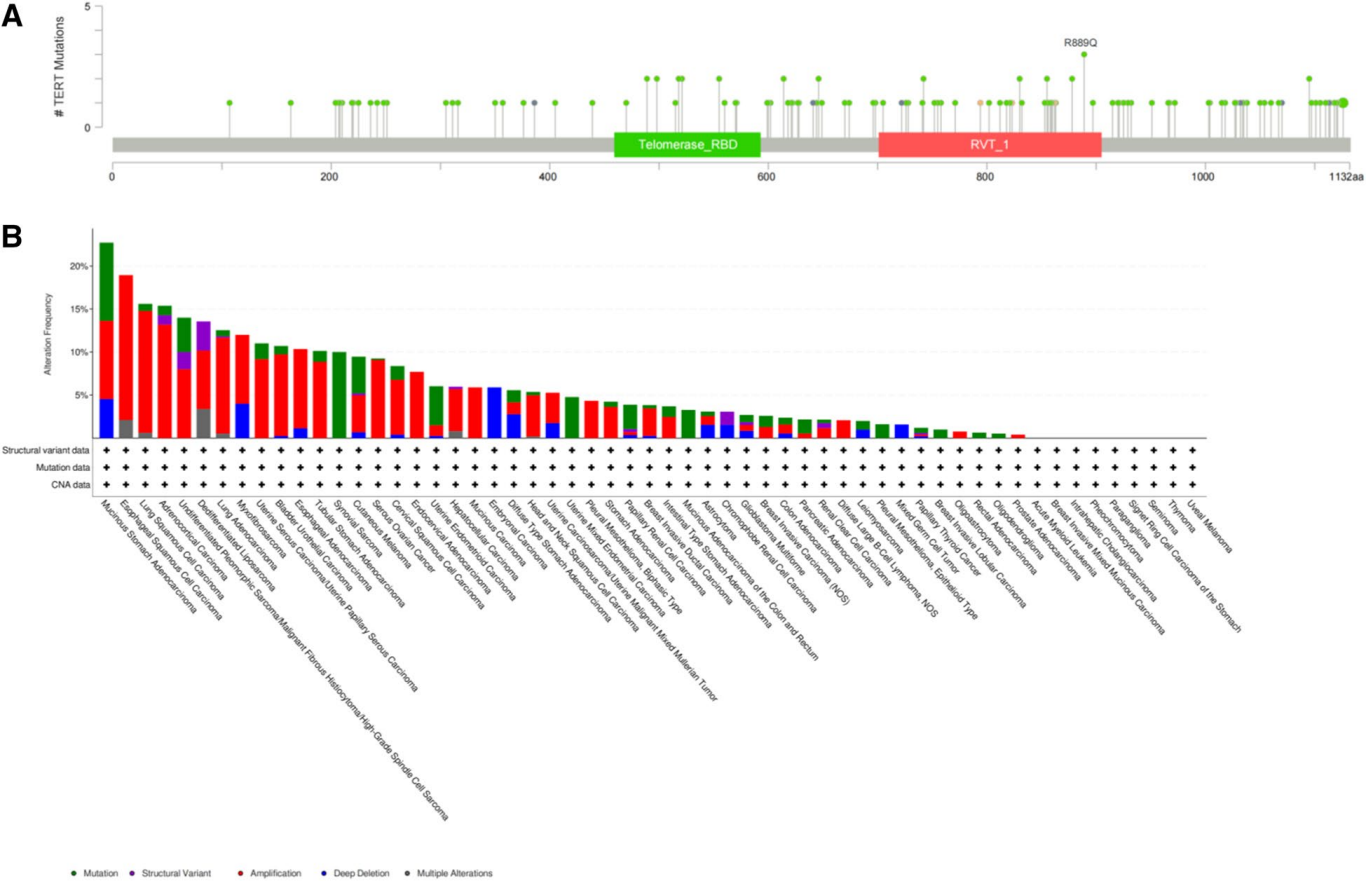
TERT gene mutation sites and mutations in pan-cancer. **A** TERT gene mutation site. **B** TERT gene mutationin in pan-cancer. The TERT gene manifests itself as mutated, deleted and enhanced in thyroid cancer

Epigenetic imbalance is an important indicator of cancer, and an abnormal methylation of cytosine residues plays an important role in abnormal gene expression in cancer cells. We also obtained the telomerase reverse transcriptase (TERT) mutation site and mutation status in different tumours through the cBioPortal (https://www.cbioportal.org/) website. TERT mutation is the R889Q point (Fig. 2A). TERT mutations can be detected in different tumours including TC (Fig. 2B). The TERT promoter of PTC and ATC exhibits significantly lower levels of 5-hmC; however, there is no significant differences were not observed the 5-hmC levels of the TERT promoter wild PTC and normal thyroid tissue. 5-hmC deficiency is an epigenetic characteristic of the methylation of the TERT promoter, which indicates that this TC exhibits evident molecular characteristics, and that the TERT promoter methylation and the BRAF^V600E^ mutation of TC indicate poor clinical prognosis [29]. Mutation of the TERT promoter is associated with highly invasive TC [30], especially in the case of a mutation in the BRAF gene [31, 32]. TERT promoter mutations have been confirmed in approximately 9% of PTC and at higher frequency in low-risk DTC (40%) and ATC (> 70%) [33]. TERT initiation mutations are common in late-stage PTC (61%) and FTC (71%) [34]. The gene encodes the catalytic subunit of telomerase, a ribonucleic complex that maintains telomere length, and plays an important role in tumour occurrence and cell immortality, and two mutation hotspots have been reported in the gene’s initiators: C228T and C250T [35, 36]. BRAF mutations and TERT promoter mutations have synergies in the diagnosis of thyroid cancer [37–42], and we have also found synergies between BRAF mutations and TERT promoter mutations in patients with melanomas [43–45], epithelial glioblastomas [46] and gliomas [47]. BRAF^V600E^ and TERT promoters at the same time mutation led to poor prognosis of thyroid papilloma cancer, which provides a certain reference value for diagnosis and treatment [48], but it still has some limitations. The specificity and sensitivity of the clinical characteristics of the reaction have yet to be further verified.

FTC can be identified by RASSF1 and TPO methylation (ROC 0.881, sensitivity 78%) and TPO and UCHL1 methylation (ROC 0.607, sensitivity 78%). Based on the PTC methylation status and normal thyroid gland identification of six genes, namely, TIMP3, RARB2, SERPINB5, RASSF1, TPO, and TSHR (ROC 0.908), 91% sensitivitywas observed in the differentiation of FTC from follicular adenoma (FA) which helps in preventing unnecessary thyroid surgery [49].

Retinoid receptors (RRs) play a key role in cell proliferation and differentiation, and they include four subtypes of RARA, RARB, RXRA, and RXRB, which are significantly more expressed in TC than follicular adenomas. High methylation of RARB2 initiators was observed in ATC patients, although methylation was not observed in thyroid tumours, PTC and FTC, thus confirming that high methylation of RARB2 promoters was associated with the malignancy of thyroid cancer and may become an effective marker for identifying ATC [50]. Studies also reported a positive correlation between RARB2 promoter methylation and the BRAF gene V600E mutation in thyroid cancer [51]. RARB2 and methylation of five other genes (TIMP3, SERPINB5, RASF1, TPO, THSHR) in the predictive model showed a sensitivity of 91%, in distinguishing between PTC and benign thyroid disease, especially for undiagnosed thyroid lesions in fine needle aspiration biopsy (FNAB), and thus can reduce unnecessary thyroid surgery [49].

Calcium binding protein-2(SMOC2) is a secreted matrix cell protein that participates in various processes related to tumour progression, such as regulating the cell cycle, angiogenesis, and invasion [52, 53]. SMOC2 is normally expressed in thyroid follicle epithelial cells, and its expression level in nodule hyperplasia remains at normal levels. However, SMOC2 is significantly reduced in lymphocytic thyroiditis and follicular tumors, including FAs and cancers. In particular, 38% of PTC exhibits a complete lack of SMOC2 expression, which is attributed to the presence of BRAF^V600E^ mutations. The results of the analysis of DNA methylation ChIP indicate that the SMOC2 gene initiator region contains a high-methylation CpG site, suggesting that SMOC2 exhibits epigenetic regulation in PTC. Note that the high expression of SMOC2 and lymph node metastasis are high risk factors for recurrence in women [54]. In summary, SMOC2 expression in PTC is significantly reduced, and the high expression of SMOC2 is closely related to a better clinical prognosis, suggesting that SMOC2 can be used as a prognostic index for PTC patients. In thyroid cancer with BRAFV600E mutations, SMOC2 expression is reduced, which suggests that the use of BRAF inhibitors may promote the expression of SMOC2 and improve the prognosis of patients.

The high methylation of the initiator of multiple genes plays a significant role in TC, most notably the high methylation of the thyroid stimulating hormone receptor (TSHR) gene initiator, which is closely related to the mutation status of the BRAF gene [55]. TSHR methylation was found in 71% of malignant nodules and 46% of benign nodules, and TSHR methylation occurred in patients with lymph node metastasis [56]. The BRAF^V600E^ mutation detection rate was 25%, the TSHR promoter methylation detection rate was 73.3%. A significant was observed between TSHR gene methylation and positive BRAF^V600E^ mutation cases (P < 0.05), suggesting that TSHR gene methylation is highly correlated with the BRAF^V600E^ mutation in thyroid tumours and that TSHR pathways are positively correlated with MAP kinase pathways [57]. TSHR expression can also be detected not only in tissues, but also in the peripheral blood of patients, and the level of TSHR mRNA expression in PTC patients is significantly higher than that in healthy populations. These studies show that thyroid disease can be diagnosed by changes in target genes in peripheral blood thyroid cells [58]. The BRAF gene regulates the growth of PTC tumour cells through TSHR [59].

DNA methylation plays an important role in the occurrence of thyroid tumours, and in different TC subtypes, different DNA methylation-specific gene changes are closely related to TC occurrence [60]. DNA methylation is a reversible process that occurs in the 5th-bit CpG dinucleotide of the cytosine ring (C5) and is mediated by the DNMT family [61]. DNMT1 is primarily involved in the maintenance of DNA methylation patterns, whereas DNMT3A and DNMT3B are responsible for methylation from scratch [62]. Methylation of the DNMT3A gene in PTC cells and recovery of DNMT3A expression through 5-aza-2ʹ-deoxycytesine demethylation enhance the expression of TRAIL-guided cell apoptosis [63].

Pleckstrin homology domain containing, family S member 1(PLEKHS1) initiator methylation is less common in PTCs and ATCs, and the high expression of PLEKHS1 is associated with PTC lymph nodes and distant metastasis but shortens OS and DFS in PTC patients. The ectopic expression of PLEKHS1 in TC cells increases the abundance of phosphorylated AKT, indicating that PI3K-AKT is involved in PTC progression mediated by PLEKHS1 [64]. The promoter mutation of PLEKHS1 was found in iodine-incurable thyroid cancer, and it can be combined with the TERT promoter, BRAF, RAS, and TP53 as classification tools to classify patients with differentiated thyroid cancer who have distal metastases [65]. PLEKHS1 demethylation induces local opening of chromosomes, which leads to the expression of PLEKHS1 in thyroid cancer. Abnormal changes in PLEKHS1 demethylation may be a target of thyroid cancer; however, the mechanism of PLEKHS1 demethylation still needs further study.

In the thyroid tissue and blood of nodular goitres, adenoma, and PTC, the degree of DNA methylation of phosphatase and tensin homologue (PTEN) and death-related protein kinase (DAPK) are negatively correlated with the transcription level, indicating that the high methylation of CpG islands in these gene initiator regions inhibits transcription, The PETN and DAPK methylation statuses combined with BRAF^V600E^ mutation improves the diagnostic performance [66]. PTEN methylation is significantly higher in ATC and FTC than in benign thyroid tumours [67]. The association between DAPK methylation and the relative odds ratio of thyroid cancer indicates that DAPK is involved in the occurrence of thyroid tumours [68]. Meanwhile, the expression of PTEN and DAPK genes are closely related to lymphatic metastasis, which indicates their relation to tumour metastasis and recurrence [66]. PTEN, as an antagonist of PI3K channels, is mainly related to cell growth and migration. The downwards expression of PTEN leads to an increase in phosphorus-AKT levels, which inhibits apoptosis [69]. It is predicted that the use of PI3K/AKT pathway inhibitors in patients with high PTEN methylation can promote apoptosis and thus achieve therapeutic effects.

P73 is a member of the p53 family and regulates cell proliferation, differentiation, and death [70]. The Wnt inhibitory factor-1 (WIF-1) gene is an antagonist that binds to Wnt protein. Moreover, it binds to Wnt to inhibit Wnt/β-catenin signalling and participates in the tumorigenesis process [71]. PDZ and LIM domain 4 (PDLIM4) is a potential tumour suppressor gene involved in cell growth regulation [72]. P73, WIF-1, and PDLIM4 gene promoters are hypermethylated and exhibit the same methylation level in the blood as that in tissues, which can be used as a diagnostic target in TC [73]. Impaired function of p73 may promote the progression of thyroid malignancies, and p73 may be a potential therapeutic target for thyroid cancer [70].

**Fig. 3:**
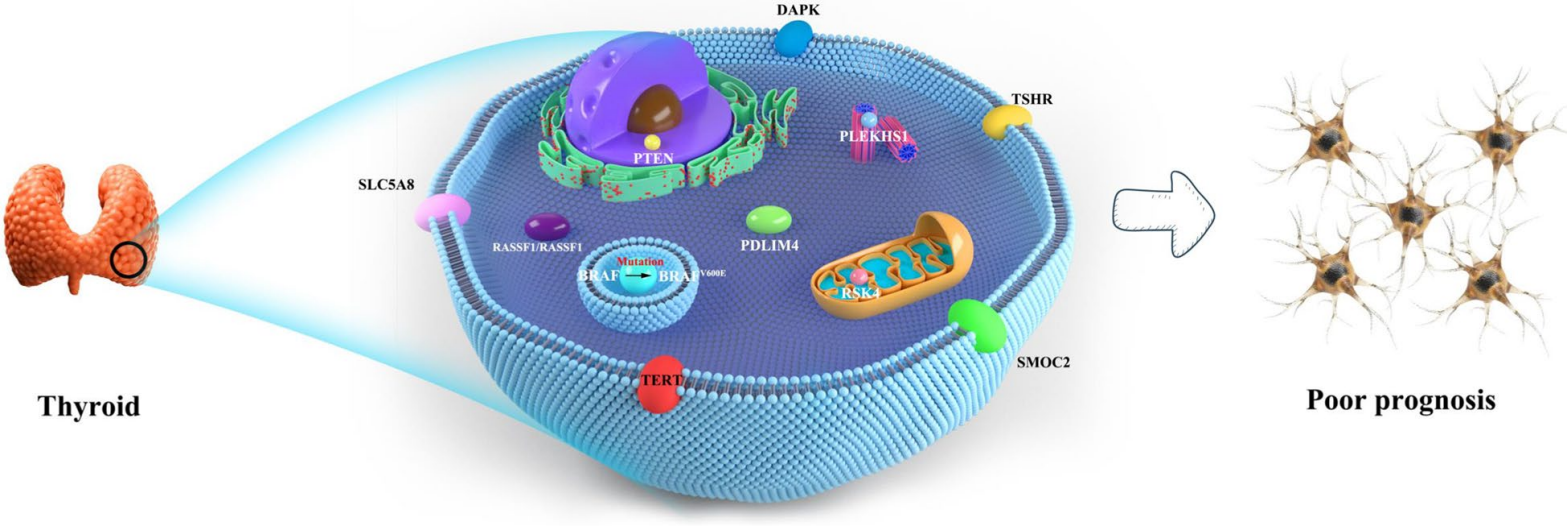
Different tumour suppressor gene methylation and BRAF gene mutation in thyroid cancer. BRAF^V600E^ is located in the 15th exon of the BRAF gene. Its mutation is the T1799A point mutation in the exon, in BRAF^V600E^ mutation, the cell can not complete the normal apoptosis, may further lead to the occurrence of tumors. In thyroid cancer, the BRAF gene usually mutates to BRAF^V600E^. The presence of promoter methylation in tumour suppressor genes (RASSF1A, SMOC2, TSHR, TERT, SLC5A8, PLEKHS1, PTEN, DAPK, PDLIM4 and RSK4) leads to decreased tumour suppressor genes expression. When the tumour suppressor genes methylation was present at the same time as BRAF^V600E^, the prognosis was poor in patients with thyroid cancer

Methylation of ribosomal protein S6 kinase (RSK4) promoters in PTC leads to a decrease in protein expression levels, while in normal thyroid tissue, the frequency of methylation of RSK4 is significantly reduced. Methylation of RSK4 promoters can lead to lymph node metastasis in patients with thyroid cancer. RSK4 expression levels decreased significantly in patients with BRAF^V600E^ mutations [74]. Interestingly, in stomach cancer, low expression of RSK4 promotes the added value and invasiveness of stomach cancer cells [75]. However, in transparent cell kidney cancer, high expression of RSK4 leads to poor prognosis [76]. Targeted inhibition of RSK4 can prevent chemotherapy resistance and metastasis of lung and bladder cancer [77], thus showing that RSK4 may have a double-edged role in tumours. High expression of RSK4 can directly act on ERK or downstream signalling, thereby inhibiting the MAPK signalling pathway and inhibiting TC. In contrast, RSK4 hypermethylation leads to the inactivation of RSK4 and activation of the MAPK pathway, which promotes TC development [74]. BRAF^V600E^ mutation abnormally activates of the MAPK pathway and plays a key role in the cancerous process of TC [78]. In addition, RSK4 may act directly on ERK to suppress the MAPK signalling pathway [79]. High RSK4 methylation and BRAF^V600E^ mutation activate the MRPK pathway to promote tumour progression.Meanwhile, RSK4 can somewhat restore its tumour suppressor effect through demethylation, which shows that targeting RSK4 demethylation drugs is an attractive alternative for the treatment of recent tumours. RSK4 demethylation restores its tumour inhibition to some extent, and RSK4 inhibitors combined with BRAF inhibitors may be a new strategy for tumour therapy [80]. The inhibition of RSK4 to MAPK pathways occurs at ERK or its downstream level and depends on the kinase activity of RSK4. RSK4 is highly expressed in thyroid tissue, and it is assumed to be an important endogenous inhibitor of MAPK pathways. RSK4 methylation may mediate the occurrence of PTC by reducing the inhibition of MAPK pathways [79]. Different tumour suppressor gene methylation and BRAF gene mutation in thyroid cancer. In thyroid cancer with BRAF gene mutations, the presence of promoter methylation in SMOC2, TSHR, TERT, SLC5A8, PLEKHS1, PTEN, DAPK, PDLIM4 and RSK4 genes would lead to poor prognosis of thyroid cancer (Fig. 3).

## Different signalling pathways and BRAF inhibitors in TC

Studies conducted on thyroid tumour cells have mainly focused on mitogen-activated protein kinase (MAPK), phosphatidylinositol 3 kinase/serine protein kinase/threonine protein kinase (PI3K/Akt), thyroid-stimulating hormone receptor/cyclic adenosine monophosphate (TSHR/cAMP), Wnt/β-catenin, and NOTCH pathways. Genetic or epigenetic changes in many genes can activate or inhibit these pathways, thereby promoting tumorigenesis [81–83].

Using BRAF inhibitors in human TC cells, the intracellular KRAS signalling pathway can be significantly enriched, including genes that are upregulated and downregulated after KRAS activation [84]. The protocarcinoma activation of the KRAS regulatory effect is attributed to the MAPK pathway [85]. In BCPAP cells treated with vemurafenib or dabrafenib, the damage caused to the BRAF^V600E^ signal on the downstream effector MEK of the MAPK pathway, as well as the inhibitory effect of its phosphorylation and feedback activation of ERK phosphorylation was detected. However, in experiments conducted with BRAF inhibitors, significant drug resistance appeared, which led to reactivation of the MAPK pathway. Activation of parallel signal cascades, such as the PI3K/AKT pathway, or acquisition of mutations in the RAS gene family leads to treatment failure [86, 87]. The BRAF inhibitor alone in TC is prone to resistance caused by reactivation of the MAPK pathway [88]. The combination of BRAF and ERK1/2 inhibitors (SCH772984) can significantly inhibit the growth of TC cells, reduce the survival rate of clone formation, enhance the apoptosis of BRAF mutant TC cells, and reduce the activity of the MARK signalling pathway. The combined use of BRAF and ERK1/2 inhibitors inhibits ERK1/2 phosphorylation and activity, thereby inhibiting the growth of TC cells. These results provide a key theoretical basis for combining BRAF and ERK1/2 inhibition as an alternative treatment strategy for patients with BRAF-mutated advanced TC [89]. In addition, the BRAF inhibitor PLX4720 activates ULK1 and induces autophagy by activating the AMPK-ULK1 pathway but not by inhibiting the mTOR signalling pathway. Blocking autophagy promotes the death of TC cells and increases the drug sensitivity of PLX4720 to BRAF-mutated TC cells. The development of therapeutic drugs for the AMPK pathway or autophagy can help improve the efficacy of BRAF inhibitors and overcome the acquired resistance of TC to BRAF inhibitors and other drugs [90].

Resistance to the RAF inhibitor PLX4032 leads to reduced benefits for patients with BRAF^V600E^ mutations in TC, mainly because the MAPK/ERK and PI3K/AKT pathways are abnormally activated by resistance. The combined use of PLX4032 and vitamin C enhances the activity of PLX4032 in the body, thereby reducing the abnormal activation of MAPK/ERK and PI3K/AKT pathways, blocking the G2/M phase of TC cells, and inducing BRAF^V600E^ mutant TC cell apoptosis [91].

The differential expression of sprouty homolog 1(SPRY1) in TPO-KRAS^G12D^ and TPO BRAF^V600E^ mice indicates that SPRY1 can selectively regulate the TSH-mediated RAS signalling pathway in TC. When its expression is down-regulated, it promotes the activation of the MAPK pathway and leads to the occurrence of DTC. In contrast, when its expression is downregulated, it promotes PI3K/AKT pathway activation and FTC initiation. The abnormal expression of RAS can escape SPRY1-mediated feedback inhibition of the MAPK signal and induce the occurrence of DTC [92].

In TC cells, the BRAF^V600E^ gene mutation and overexpression of the NOTCH pathway domain caused the mouse tumour to grow faster and more invasively, resulting in a decline in overall survival. The BRAF^V600E^ MTC cell line is prone to resistance to NOTCH pathway inhibitors mainly because the BRAF^V600E^ mutation may bypass the NOTCH pathway and significantly enhance its downstream targets in TC, Hes family bHLH transcription factor 1 (HES-1) and HEY-1, thereby promoting tumorigenesis [93]. Abnormal localization of β-catenin exists in many human cancers, including TC. Up to 90% of PTCs have demonstrated abnormal localization of nuclear and cytoplasmic β-catenin through immunohistochemistry [94]. The β-catenin protein is highly expressed in PTC, and confirms the activation of the Wnt pathway, which increases the expression of β-catenin protein. Nonsteroidal antiinflammatory drugs (NSAIDs) can reverse the activity of this protein and inhibit the expression of cyclin D1, matrix metalloproteinases, c-myc, and other oncogenic factors, thereby reducing the invasion ability of BRAF^V600E^ mutant tumours [95].

**Fig. 4:**
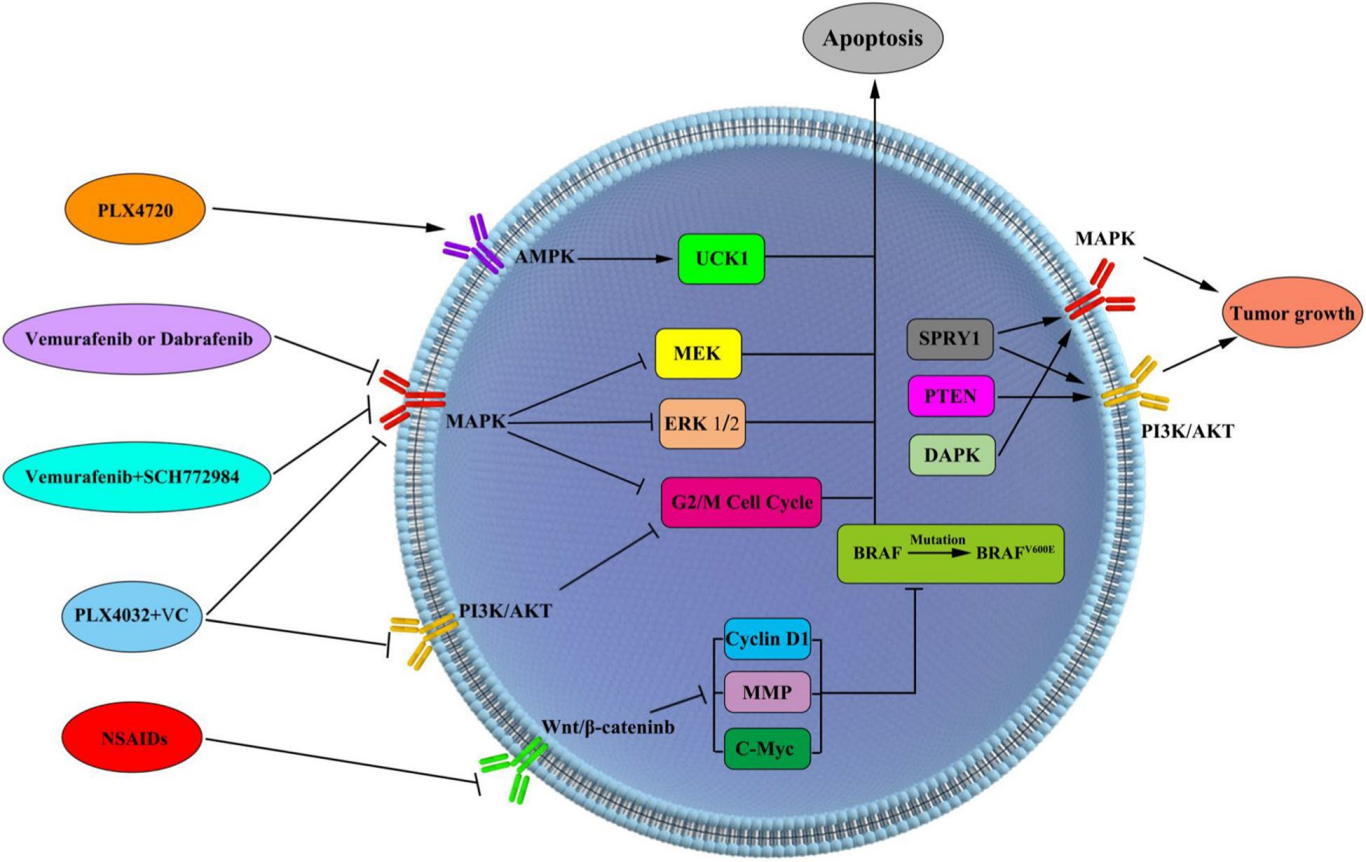
Different signaling pathways and BRAF inhibitors in thyroid cancer. BRAF and ERK1/2 inhibitors (SCH772984) reduce the activity of the MARK signaling pathway and inhibit ERK1/2 phosphorylation and activity, thereby inhibiting the growth of TC cells. BRAF inhibitor PLX4720 activates ULK1 by activating AMPK-ULK1 and inhibits autophagy, which promotes the death of TC cells. PLX4032 and vitamin C enhances the activity of PLX4032 in the body, thereby reducing the abnormal activation of MAPK/ERK and PI3K/AKT pathways, blocking the G2/M phase of TC cells, and inducing BRAFV600E mutant TC cell apoptosis. NSAIDs can reverse the activity of this protein and inhibit the expression of cyclin D1, matrix metalloproteinases, c-myc, and other oncogenic factors, thereby reducing the invasion ability of BRAFV600E mutant tumors. SPYR1 activates the MAPK/PI3K/AKT pathway to promote tumour cell growth. The hypermethylation of the PTEN and DAPK inactivating the P13K/Akt and MAPK signaling pathway promoted TC cell growth

The ten-eleven translocation (TET) protein DNA demethylase has been identified, and the TET protein family comprises three members: TET1, TET2, and TET3 [96]. TET protein can catalyse the conversion of 5-methylcytosine (5mC) into 5-hydroxymethylcytosine (5hmC), followed by formylcytosine (5fC) and 5-carboxycytosine pyrimidine (5caC) [97]. DNA methylation is a dynamic process that affects the distribution of cytosine forms (5hmC, 5fC, and 5caC) in the entire genome. The 5mC level is related to transcriptional silencing and is usually found in repetitive and heterochromatic regions of the genome, whereas 5hmC is rich in promoter regions, open-reading frames, and intergenic regions and associated with the gene transcription activity [98, 99]. High TET1 and TET2 methylation in PTC led to low presentation levels and decreased TET1 expression levels in patients with BRAF gene mutations. The TET2 gene is found in thyroid cancer, especially in PTC samples. BRAF inhibitors may be used to treat patients with high methylation of TT1 and TT2 [100]. At the same time, the treatment of colon cancer was unsuccessful in colorectal cancer through BRAF-mediated TET silencing [101]. As a tumour suppressor gene, PTEN is inactivated by point mutations, gene deletions, posttranslational modifications and epigenetic silencing caused by hypermethylation of its promoter. The epigenetic modification of PTEN plays an important role in the occurrence of TC. DAPK is involved in cell growth, apoptosis and autophagy. DAPK hypermethylation leads to gene silencing, and combined with BRAF mutations, leading to the occurrence and progression of PTC tumoyrs [102]. Hypermethylation of the PTEN promoter is common in thyroid tumors, which leads to abnormalities in the PI3K/Akt pathway promoted by PTEN [67]. The hypermethylation of DAPK leads to inactivation of the MAPK signalling pathway. Due to the important roles of PTEN and DAPK in early TC diagnosis, cancer screening, and treatment prospects, they are assigned the methylation status of a biomarker [68, 103]. Different signaling pathways and BRAF inhibitors in thyroid cancer. MAPK signal pathways, PI3K/Akt signaling pathways, TSHR/cAMP signaling pathways, Wnt/β-cateninb signaling pathways, and AMPK signaling pathways are involved in epigenetic changes in thyroid cancer with BRAF gene mutations, activating or inhibiting these pathways affecting the development of thyroid cancer (Fig. 4).

## Histone modification and BRAF gene mutation in TC

**Fig. 5:**
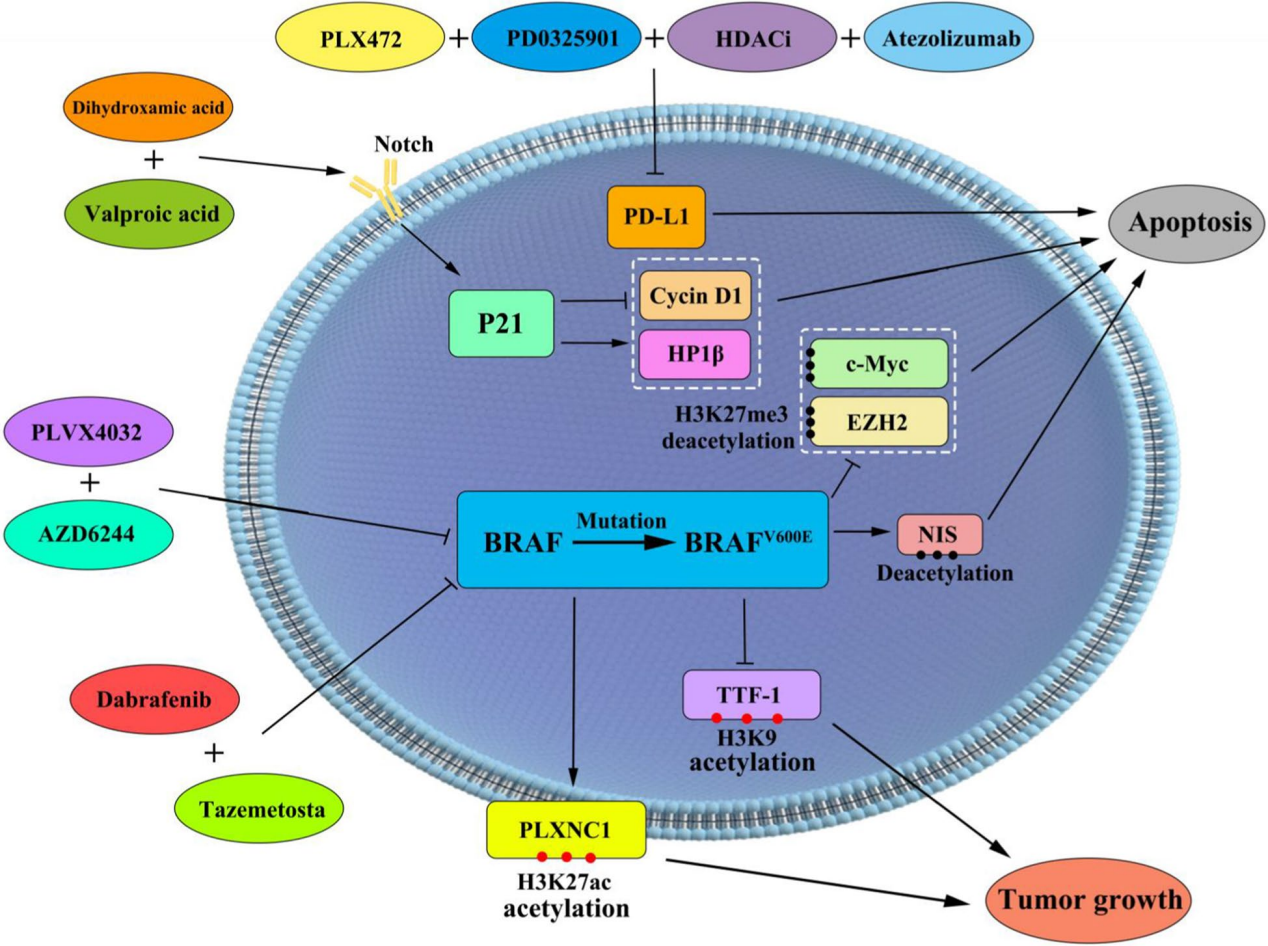
Histone modification inhibitors and BRAF inhibitors in thyroid cancer. The combination of Dabrafenib and Tazemetostat inhibits c-Myc and EZH2 protein activity through H3K27me3 deacetylating, promoting the apoptosis of BRAFV600E mutant PTC cells. PLX4032 and AZD6244 increase the histone acetylene of NIS promoters, leading to apoptosis of thyroid cancer cells. Dihydroxamic acid and VA can increase the expression of HP1β by activating the Notch signaling pathway, increasing the expression of cyclin-dependent kinase inhibitor P21, and decreasing cyclin D1, which inhibits the growth of TC cells. HDACi combined PLX472, PD0325901 and atzolizumab inhibited PD-L1 expression for the treatment of BRAF mutations in thyroid cancer cell immunotherapy. BRAF mutations inhibit H3K9 acetylation of TTF-1 and promoting acetylation of H3K27ac in PLXNC1 then promote the proliferation of thyroid cancer cells

The BRAF^V600E^ mutation activates the MAPK/ERK signalling pathway, which can control cell growth and survival through epigenetic modifications (including DNA methylation and histone modifications). Histone is the main protein element of chromosome structure. There are many kinds of posttranslation modifications of histones, including methylation, acetylation, phosphorylation, and ubiquitination [104]. These modifications can affect the interaction between DNA and histones, leading to changes in gene transcription, DNA repair, DNA replication, and chromosome arrangement [105–108]. Compared to DTC, the level of acetylated H3K18 is lower in undifferentiated TC, and this transition can be caused by the closure of acetylation. This result indicates that histone acetylation is an early event in the prognosis of TC [13]. Hypermethylation of thyroid transcription factor-1 (TTF-1) reduces H3K9 acetylation and increases H3K9 methylation, downregulating the expression of TTF-1 and leading to the occurrence of TC [109]. In addition, the enhancer of histone lysine methyltransferase Zeste homologue 2 (EZH2) belongs to the polycomb histone family, which is significantly upregulated in ATCs and directly induces TC differentiation. The increase in H3K27me3 caused by BRAF^V600E^ mutant PTC cells can be achieved by enhancing the expression of c-Myc and EZH2. The combination of MAPK inhibitor and tazemetostat inhibits the expression and protein activity of EZH2, which significantly decreases downstream H3K27me3 and effectively promotes the differentiation of BRAF^V600E^ mutant PTC cells [110]. Compared to normal tissues, the acetylation of lysine 9–14 on histone H3 in TC and the expression level of oncogenes are higher [104]. Histone acetylation is closely related to transcriptional activity, and deacetylation can induce transcriptional silencing. Histone modifications control gene expression, and their dysregulation functionally affects the carcinogenic effects of the transcriptome. The histone deacetylation inhibitor vorinostat can induce PTC cell apoptosis and is used in clinical trials for TC treatment [111]. The BRAF^V600E^ mutation leads to impaired expression of the sodium iodide symporter (NIS) and resistance to radioiodine therapy in TC, which is mainly because BRAF^V600E^ enhances overall histone acetylation and inhibits histone deacetylation at the NIS promoter. The BRAF^V600E^ inhibitor PLX4032 and MEK inhibitor AZD6244 increase the histone acetylation of the NIS promoter, indicating that BRAF^V600E^ normally maintains histone deacetylation at the NIS promoter [112]. Histone deacetylase is overexpressed in tumours, thus blocking the transcription of suppressor genes to promote tumour progression [113]. Histone deacetylase inhibitors (HDACi), which can block tumour cell growth and promote cell apoptosis, are currently being tested in clinical trials. In TC, HDACi induces cell death by activating cysteine proteases and downregulating BCL2 expression. In addition, HDACi valproic acid (VA) enhances the sensitivity of tumour cells to chemotherapy, radiotherapy, and surgery [114]. The high expression level of heterochromatin protein 1β (HP1β), which can reduce the infiltration and metastasis of cancer cells, decreases in the advanced stage of TC. The HDACi suberoyl dihydroxamic acid and VA can increase the expression of HP1β by activating the Notch signalling pathway, increasing the expression of cyclin-dependent kinase inhibitor P21, and decreasing cyclin D1, which inhibits the growth of TC cells [115]. By applying H3K27ac chromatin immunoprecipitation to TC tissue and benign thyroid nodules, followed by deep sequencing (ChIP-seq) and RNA sequencing, a comparison of the epigenomic characteristics of the two indicates that H3K27ac expression in TC tissue is significantly higher than that in benign thyroid nodules. Meanwhile, changes are detected in H3K27ac levels in the active regulatory region, and the PTC-specific superenhancer Plexin C1 (PLXNC1), which is involved in the immune response and cancer-related pathways, has been shown to affect the disease-free survival rate of PTC [116]. BRAF(PLX4720) or MEK kinase inhibitors(PD0325901) exhibit a certain inhibitory effect on the survival, proliferation, and migration of tumour cells. The combined application of the two drugs inhibits the survival, proliferation, and migration of TC cells but cannot induce cell death. HDACi (suberoylanilide hyroxamic acid, SAHA) can cause cell cycle arrest but does not affect cell migration. Therefore, the combination of HDACi and the two drugs can significantly induce PD-L1 expression for immunotherapy and cause the death of primary BRAF-mutated TC cells [117]. Inhibiting the activity of lysine methyltransferase 5A (KMT5A) can inhibit the G1/S phase of PTC cells, and the increase in the KMT5A expression level is closely related to lymph node metastasis of PTC [118]. Histone modification is an important field of epigenetic research, and the abnormal expression and impaired function of histones are inseparable from the occurrence and development of TC. The in-depth study of histone modification is not only conducive to the elucidation of the TC mechanism but also provides new treatment strategies for developing new targeted drugs for treating TC. Histone modification inhibitors and BRAF inhibitors in thyroid cancer. BRAF inhibitors, histone modification inhibitors, MEK and MAPK inhibitors promote apoptosis of thyroid cancer cells. Histone modification TTF1 and PLXNC1 promotes the progression of thyroid cancer (Fig. 5).

## RNA methylation in TC

The mammalian transcriptome contains many RNA molecules that do not encode proteins.In fact, the quantitative proportion of noncoding RNA to mRNA exceeds 10:1. In addition to DNA methylation and histone modification, noncoding RNAs also play an important regulatory role in epigenetic regulation [119]. Among them, the study is more long noncoding RNAs [120], microRNAs [121] and m6A modifications have been studied [122]. These findings suggest that specific miRNAs play a key role in the progression and development of PTC. Among them, lncRNAs play an important role in the physiology of TC cells [123]. lncRNAs directly bind to target proteins and undergo posttranscriptional modification in various malignant tumours, and different lncRNAs participate in the occurrence and development of TC through histone modification. In TC, the ncRNA BRAF-activated non-protein coding RNA (BANCR) activated by BRAF is closely related to the BRAF^V600E^ mutant, and the BANCR produced by the BRAF^V600E^ mutation is also related to the occurrence of thyroid tumours [124]. EZH2 is an oncogenic histone methyltransferase and a well-known histone modifier. The expression of BANCR in PTC tissues is significantly higher than that in neighbouring tissues. EZH2 can recruit BANCR to increase the expression level of TSHR and promote the proliferation of IHH-4 TC cells. Silencing BANCR can reduce EZH2 chromatin recruitment and TSHR expression and inhibit the proliferation of TC cells [125]. Long noncoding RNA-LUCAT1 inhibits the cyclin-dependent kinase 1(CDK1)/EZH2 axis, promotes the expression of HDAC1, inhibits the epigenetic modification of DNMT1, promotes the expression of the P53/BAX axis, and regulates the development of TC by participating in cell cycle regulation, apoptosis, and proliferation [126]. lncRNA PVT1 is highly expressed in TC tissues and cells, and its expression level is related to the TNM staging of TC and lymph node metastasis. The expression level of PVT1 in patients with tumour-infiltrated lymph node metastases is significantly higher than that in patients without the characteristics of these aggressive diseases. lncRNA PVT1 enhances the expression of the insulin-like growth factor 1 receptor (IGF1R) by competitively combining with miR-30a, thus promoting the development of PTC^[^ [127]. IGF1R plays a role in maintaining normal thyroid morphology, and elevated TSH promotes thyroid nipple proliferation when IGF1R signals are missing [128]. In approximately 5% of thyroid cancers, overexpression of insulin-like growth factor 2 binds to protein 3 (IGF2BP3) and low expression of IGF1R inhibit the abnormal growth of tumour cells caused by IGF2BP3 [129]. The acquired resistance to BRAF inhibitors is maintained by IGF1R-driven tumour vascular reconstruction [130]. Silencing the m6A methyltransferase like 3 (METTL3) can inhibit the migration ability and Wnt activity in TPC-1 cells. Upregulated METTL3 promotes TCF1 methylation and TC progression [18]. In a study of drug resistance in ATCs,, miR-27b-3p downregulated the protein and mRNA levels of PPARγ, and the overexpression of PPARγ increased the sensitivity of SW1736/Dox and 8305C/Dox cells to adriamycin. miR-27b-3p/PPARγ facilitates the resistance of human ATC cells to adriamycin, suggesting that the targeted inhibition of miR-27b-3p can help overcome the resistance of ATC cells [131]. The expression of miR-219-5p in PTC patients is related to sex, tumour size, and lymph node metastasis. The overexpression of miR-219-5p can inhibit the proliferation and migration of PTC cells and promote their apoptosis. Further studies have shown that estrogen receptor alpha (ERα) is the direct target of miR-219-5p, which mediates the role of miR-219-5p in PTC occurrence. MiR-219-5p expression is negatively correlated with Era expression. The overexpression of ERα in PTC cells promotes the effect of miR-219-5p on the proliferation and migration of PTC cells, indicating that miR-219-5p has a negative regulatory effect on PTC occurrence by targeting ERα [132].

## Conclusions

Epigenetic is an emerging field of biology, and epigenetic modification, as an important part of epigenetics, provides a new perspective for exploring the pathogenesis of TC. According to the literature, epigenetic modification exhibits a complex relation with TC and plays an important role in the occurrence and development of TC. Such findings will facilitates the discovery of new biological markers for detecting TC.

However, shortcomings are observed in fully applying epigenetics in the clinic. (1) Most clinical studies are cross-sectional studies or preliminary trials, in which epigenetic modification is understood more at the theoretical level. Clinical studies mainly discuss thyroid cancer in terms of DNA methylation; thus, research on thyroid cancer is relatively limited in direction. Moreover, epigenetic modifications are usually interactions, and current experiments are mostly limited to studying the effect of a certain epigenetic modification on TC. Thus, the effects of epigenetic modification on TC are diversified. (2) Both genetic variation and epigenetic modification play key roles in the occurrence and development of TC. Although epigenetic changes can be reversed under certain conditions, but the relation between the two is currently poorly understood. (3) TC has a good prognosis and is mostly treated by surgical approaches; however, surgical trauma significantly impacts the human body. Recently, new histone inhibitors have been applied in clinical trials, and they have successfully improved the prognosis of high-grade TC. Targeted drugs can provide preventive treatment after the patient is diagnosed with TC, reduce the tumour’s malignancy and metastasis, and prevent the need for surgery. (4) Further research is required to clarify the known epigenetic modifications and explore the pathogenesis of TC. In addition, various testing tools and techniques, such as bioinformatics and high-throughput sequencing, need to be used to strengthen the relevant basic and clinical research and clarify the role of epigenetic modification in TC pathogenesis, thus paving the way for disease prevention, diagnosis, and treatment and the development of new drugs.

## Data Availability

All data in this study can be obtained by contacting the corresponding author.

## Abbreviations

TC: Thyroid carcinoma
PTC: Thyroid papillomavirus
FTC: Follicular thyroid cancer
DTC: Differentiated thyroid cancer
MTC: Myelin-Like thyroid cancer
ATC: Anaplastic thyroid cancer
LDTC: Low-differentiated thyroid cancer
UC: Undifferentiated thyroid carcinomas
FA: Follicular adenomas
DNMT: DNA methyltransferase
DNMT3A: DNA methyltransferase 3 alpha
DNMT3B: DNA methyltransferase 3 beta
DNMT3L: DNA methyltransferase 3 like
SLC34A2: Solute carrier family 34 (type II sodium/phosphate cotransporter), member 2
EMT: Epithelial-mesenchymal transitions
HATS: Histone acetyltransferase
HDACs: Histone deacetylation enzymes
KDM5C: Lysine (K)-specific demethylase 5C
TRPM3: Transient receptor potential cation channel, subfamily M, member 3
TCF1: HNF1 homeobox A
RASSF1A: RAS-associated domain family protein 1
SLC5A8: Solute carrier family 5 (sodium/monocarboxylate cotransporter), member 8
TERT: Telomerase reverse transcriptase
TPO: Thyroid peroxidase
UCHL1: Ubiquitin carboxyl-terminal esterase L1
RRs: Retinoid receptor
RARA: Retinoic acid receptor, alpha
RARB: Retinoic acid receptor, beta
RXRA: Retinoid X receptor, alpha
RXRB: Retinoid X receptor, beta
RARB2: Retinoic acid receptor, beta 2
FNAB: Fine needle aspiration biopsy
SMOC2: Calcium binding protein-2
TSHR: Thyroid stimulating hormone receptor
PLEKHS1: Pleckstrin homology domain containing, family S member 1
PTEN: Phosphatase and tensin homolog
DAPK: Death-related protein kinase
WIF-1: Wnt inhibitory factor-1
PDLIM4: PDZ and LIM domain 4
RSK4: Ribosomal protein S6 kinase
MAPK: Mitogen-activated protein kinase
HES-1: Hes family bHLH transcription factor 1
NSAIDs: Nonsteroidal antiinflammatory drugs
TET: The ten-eleven translocation
TET1: Tet methylcytosine dioxygenase 1
TET2: Tet methylcytosine dioxygenase 2
TTF-1: Thyroid transcription factor-1
EZH2: Enhancer of histone lysine methyltransferase Zeste homolog 2
NIS: Sodium iodide symporter
HDACi: Histone deacetylase inhibitors
VA: Valproic acid
HP1β: Heterochromatin protein 1β
PLXNC1: Plexin C1
KMT5A: Lysine methyltransferase 5A
BANCR: BRAF-activated non-protein coding RNA
CDK1: Cyclin-dependent kinase 1
IGF1R: Insulin-like growth factor 1 receptor
METTL3: Methyltransferase like 3
ERα: Estrogen receptor alpha
SPRY1: Sprouty homolog 1
